# Cardiovascular magnetic resonance left ventricular strain in end-stage renal disease patients after kidney transplantation

**DOI:** 10.1186/s12968-018-0504-5

**Published:** 2018-12-17

**Authors:** Inna Y. Gong, Bandar Al-Amro, G. V. Ramesh Prasad, Philip W. Connelly, Rachel M. Wald, Ron Wald, Djeven P. Deva, Howard Leong-Poi, Michelle M. Nash, Weiqiu Yuan, Lakshman Gunaratnam, S. Joseph Kim, Charmaine E. Lok, Kim A. Connelly, Andrew T. Yan

**Affiliations:** 1 University of Toronto, Toronto, Canada; 2 Department of Medical Imaging, St Michael’s Hospital, Toronto, Canada; 3 Keenan Research Centre, Li Ka Shing Knowledge Institute, St. Michael’s Hospital, Toronto, Canada; 4 Terrence Donnelly Heart Centre, St. Michael’s Hospital, Toronto, Canada; 5 Division of Nephrology, St Michael’s Hospital, Toronto, ON Canada; 6 Division of Cardiology, Toronto General Hospital, Toronto, Canada; 7 Division of Nephrology, Department of Medicine, London Health Sciences Centre, Schulich School of Medicine and Dentistry, Western University, London, Canada; 8 Department of Medicine, Division of Nephrology, Toronto General Hospital, University Health Network, Toronto, Canada; 9 Department of Medicine, University Health Network-Toronto General Hospital, Toronto, Canada; 10 Division of Cardiology, St. Michael’s Hospital, 30 Bond Street, Rm 6-030 Donnelly, Toronto, M5B 1W8 Canada

**Keywords:** Kidney transplant, Cardiovascular magnetic resonance, Left ventricular peak systolic strain, Left ventricular ejection fraction, Left ventricular volume

## Abstract

**Background:**

Cardiovascular disease is a significant cause of morbidity and mortality in patients with end-stage renal disease (ESRD) and kidney transplant (KT) patients. Compared with left ventricular (LV) ejection fraction (LVEF), LV strain has emerged as an important marker of LV function as it is less load dependent. We sought to evaluate changes in LV strain using cardiovascular magnetic resonance imaging (CMR) in ESRD patients who received KT, to determine whether KT may improve LV function.

**Methods:**

We conducted a prospective multi-centre longitudinal study of 79 ESRD patients (40 on dialysis, 39 underwent KT). CMR was performed at baseline and at 12 months after KT.

**Results:**

Among 79 participants (mean age 55 years; 30% women), KT patients had significant improvement in global circumferential strain (GCS) (*p* = 0.007) and global radial strain (GRS) (*p* = 0.003), but a decline in global longitudinal strain (GLS) over 12 months (*p* = 0.026), while no significant change in any LV strain was observed in the ongoing dialysis group. For KT patients, the improvement in LV strain paralleled improvement in LVEF (57.4 ± 6.4% at baseline, 60.6% ± 6.9% at 12 months; *p* = 0.001). For entire cohort, over 12 months, change in LVEF was significantly correlated with change in GCS (Spearman’s *r* = − 0.42, *p* < 0.001), GRS (Spearman’s *r* = 0.64, *p* < 0.001), and GLS (Spearman’s *r* = − 0.34, *p* = 0.002). Improvements in GCS and GRS over 12 months were significantly correlated with reductions in LV end-diastolic volume index and LV end-systolic volume index (all *p* < 0.05), but not with change in blood pressure (all *p* > 0.10).

**Conclusions:**

Compared with continuation of dialysis, KT was associated with significant improvements in LV strain metrics of GCS and GRS after 12 months, which did not correlate with blood pressure change. This supports the notion that KT has favorable effects on LV function beyond volume and blood pessure control. Larger studies with longer follow-up are needed to confirm these findings.

**Electronic supplementary material:**

The online version of this article (10.1186/s12968-018-0504-5) contains supplementary material, which is available to authorized users.

## Introduction

Chronic kidney disease (CKD) is well-known risk factor for adverse cardiovascular events [1]. Despite advances in dialysis and kidney transplant (KT), patients with end-stage renal disease (ESRD) and KT continue to experience high cardiovascular morbidity and mortality, even following KT [2–4].

While left ventricular (LV) hypertrophy (LVH) has been identified as a marker of poor prognosis and adverse outcomes in dialysis patients, a large proportion of ESRD patients have preserved LV ejection fraction (LVEF) [5–7]. However, measurable reduction in LVEF represents late LV dysfunction, and may only identify CKD patients with well-established cardiovascular disease [8]. Although structural changes such as LV mass (LVM) and volume have been associated with subsequent reduction in LVEF, LV myocardial deformation (strain) is likely a more sensitive measure of early subclinical myocardial dysfunction as it directly reflects the motion of myocardial fibers. Indeed, strain has emerged as a marker of LV function, and its role has been studied in a variety of heart diseases, providing incremental prognostic information beyond LVEF [9–12]. The most well-established strain parameter is global longitudinal strain (GLS), which is more sensitive than LVEF for detection of subclinical LV dysfunction [9, 12]. Given that LV strain is less load dependent, its use to evaluate changes in LV function is particularly attractive in ESRD patients who are subject to large fluctuations in preload and afterload [13]. Indeed, previous studies have shown that LV strain is likely a better measure of systolic function than LVEF in ESRD [14, 15].

Prior studies have demonstrated improved LVEF and regression of LVM post-KT [16, 17], but insufficient data exist for evaluating the impact of KT (the most effective form of renal replacement therapy) on LV myocardial function beyond changes in loading conditions. Accordingly, our study aimed to address whether KT improves systolic function as measured by LV strain, beyond volume and blood pressure control. To this end, we conducted an observational cohort study to compare cardiovascular magnetic resonance imaging (CMR)-derived changes in LV strain over 12 months between ESRD patients who underwent KT and those who remained on dialysis. There are also a paucity of data delineating the relationships between changes in myocardial strain (function), and LV remodeling (structure), in the setting of KT. As CMR is the gold standard for examining both cardiac structure (LVM and LV volumes) and function, it is of particular interest to evaluate whether a structure-function relationship exists in this patient population. Accordingly, we also examined the relationships between LV strain and other cardiac parameters including LVEF, LVM, and LV volumes.

## Methods

### Study design

The full details of the study design have been described previously [18]. Briefly, we conducted an observational cohort study of adult patients on hemodialysis or peritoneal dialysis who were single-organ KT candidates at three academic dialysis and KT centers in Ontario, Canada: St. Michael’s Hospital and Toronto General Hospital, both in Toronto, Ontario and London Health Sciences Centre in London, Ontario between August 30, 2010 and February 14, 2014.

The study was approved by the Research Ethics Boards at all study sites and all study participants provided informed consent. The inclusion criteria were: age ≥ 18 years old, approved or likely to be approved for a KT (living donor or deceased donor wait list), renal replacement with hemodialysis or peritoneal dialysis, living donor recipients at low immunological risk for graft rejection, and ability to provide informed consent.

Exclusion criteria included multi-organ transplant, pre-dialysis, daily hemodialysis, high immunological risk as per the site investigator, unlikely to receive transplant, acute coronary syndrome or coronary revascularization procedure within 6 months of enrollment, heart failure, permanent atrial fibrillation, pregnancy or intention to pursue pregnancy within 12 months, and a life expectancy < 1 year.

Patients who met the study entry criteria were separated into two groups based on availability of a potential living kidney donor. The KT group included dialysis patients who were expected to receive a living donor KT in the subsequent two months. The dialysis group comprised patients who were eligible for KT but had no living donors and were expected to remain on dialysis for the following 24 months.

Blood pressure was measured using a validated automated device according to American Heart Association Guidelines.

### CMR image processing

Baseline CMR was performed following recruitment (i.e. after enrollment and prior to KT), followed by repeat CMR at 12 months post-transplant or post-recruitment for the dialysis group. If a patient in the dialysis group unexpectedly received a KT within 12 months, the second CMR was performed 12 months after the original CMR. For hemodialysis patients, CMR was performed following dialysis to minimize effects of intravascular volume shifts. All CMR examinations were performed with a 1.5 T scanner (Intera, Philips Healthcare, Best, The Netherlands, or a GE Signa Excite Cv, Milwaukee, Wisconsin, USA) using a cardiac coil and retrospective electrocardiographic gating. The Philips 1.5 T scanner used a 5-channel (SENSE) cardiac coil. One GE 1.5 T scanner used a 32-channel cardiac coil, while another GE 1.5 T scanner used an 8-channel cardiac coil. Standard protocols using validated, commercially available sequences were used. Images were obtained with breath-hold at end-expiration. Typical balanced steady-state free precession sequence (bSSFP) parameters were used to acquire cine images in long axis planes followed by sequential short-axis cine loops with the following parameters: repetition time 4 ms, time to echo 2 ms, slice thickness 8 mm, field of view 320–330 × 320-330 mm (tailored to achieve optimal spatial resolution and image acquisition time), matrix size 256 × 196, temporal resolution of < 40 ms (depending on the heart rate) and flip angle 50 degrees. Prior to imaging processing, all CMR studies were de-identified and assigned a unique identification code. CMR studies were analyzed with commercially available cvi42 software (Circle Cardiovascular, Calgary, Canada). An experienced reader measured LVEF and LVM, while another experienced reader independently performed LV strain analysis. Readers were blinded to patient group (KT versus dialysis patients) and timing of the CMR (baseline versus 12 months).

LV end-diastolic volumes (EDV) and end-systolic volume (ESV) were measured using the short-axis cine images by manually tracing endocardial contours during end-diastole and end-systole, using the blood volume method, including papillary muscles and trabeculations. LVEF was calculated as (LVEDV-LVESV)/LVEDV × 100%. LVM was calculated using the area occupied between the endocardial and epicardial borders multiplied by the slice thickness and interslice distance, using contiguous short-axis slices at end-diastole [19]. LVEDVi, LVESVi, and LVMi were normalized (indexed) by dividing their values by the subject’s body surface area.

LV strain imaging was performed using feature-tracking (FT) CMR according to previously published methods [20]. Endocardial and epicardial borders were manually drawn in the end-diastolic frame, which were then automatically propagated (tracked) throughout the cardiac cycle. The peak systolic strain was derived from the distance moved between frames. Systolic strain is the percent change in length relative to baseline length (Langrangian strain); a positive strain implies elongation while negative strain implies shortening (e.g., a negative change in GLS from baseline to 1-year means improved function) [21]. The peak systolic LV strain parameters calculated were GLS, global circumferential strain (GCS), and global radial strain (GRS). Multiple 2D long-axis cine images (2, 3, and 4-chamber views) were tracked to derive GLS, while short-axis cine images were used to derive GCS and GRS. Strain was obtained for each segment and the global values were defined as the mean of all segmental values.

### Biomarkers

N-Terminal - brain natriuretic peptide (NT-BNP) was measured using the Cobas 6000 601e assay (Roche, Mississauga, Ontario, Canada). We also measured the growth differentiation factor-15 (GDF-15), a novel biomarker expressed in response to tissue injury with elevations implicated in worsening kidney function among patients with CKD [22], using Quantikine ELISA assay (R&D Systems Inc., Minneapolis, Minnesota, USA).

### Statistical analysis

Continuous data are expressed as mean with standard deviation or median with interquartile range (IQR), as appropriate. The Student’s *t*-test was used for normally distributed continuous data while the Kruskal–Wallis test was used for non-normally distributed continuous data. Chi-square or Fisher’s exact test was used to compare categorical variables between groups. For within-group comparisons between the baseline and 12-month follow-up CMR parameters, a paired t-test was used. The relationships between change in LV peak systolic strain parameters with changes in LVEF, LVMi, LVEDVi, LVESVi, blood pressure, dialysis vintage, and renal function as measured by estimated glomerular filtration rate (eGFR) and creatinine level at 12 months were examined using non-parametric Spearman’s correlation test. The relationships between baseline NT-BNP, GDF-15, and c-reactive protein (CRP) with LV strain parameters were examined using non-parametric Spearman’s correlation test. To determine intra-observer reproducibility, a random sample of 20 CMRs were measured by the same reader in 6 months, and intra-class correlation coefficients for absolute agreement were calculated. Statistical significance was defined as a two-sided *p* value < 0.05. All data were analyzed using SPSS version 22 (International Business Machines Corp., Armonk, New York, USA).

## Results

We consented 89 patients of whom 79 (22 peritoneal dialysis and 57 hemodialysis; 40 patients continued on dialysis and 39 patients received KT) had complete CMR-derived measurements at baseline and at 12 months (Table 1). Incomplete CMR were due to post-transplant graft failure and patient reluctance to undergo a second CMR. Two patients crossed over from the dialysis control group to the KT group due to receipt of KT from a deceased donor sooner than anticipated, and included in the KT group for analysis. One patient in the KT group underwent arteriovenous fistula closure during 12-month follow up. Prior to baseline CMR, the median (interquartile range [IQR]) dialysis vintages of patients in the dialysis and KT group were 24 (15–42) and 14 (9–28) months, respectively. We found no significant correlation between dialysis vintage and baseline CMR parameters (all *p* > 0.05, data not shown).

**Table 1:** Baseline characteristics of kidney transplant and dialysis patients

Characteristic	Dialysis patients (*n* = 40)	Kidney transplant patients (*n* = 39)	*P* value
Age, years, mean (s.d.)	56 (11)	47 (12)	0.001
Sex, male	28 (70)	27 (69)	0.57
BMI, kg/m^2^, mean (s.d.)	26.7 (4.8)	26.0 (4.6)	0.60
Cardiovascular risk factors, *n* (%)			
Hypertension	37 (93)	36 (92)	0.65
Diabetes	17 (43)	11 (28)	0.14
Dyslipidemia	34 (85)	27 (69)	0.080
Current smoking	4 (10)	2 (5.1)	0.68
Cardiovascular disease			
Myocardial infarction	4 (10)	2 (5.1)	0.35
Stroke	3 (7.5)	0 (0)	0.13
Heart failure	2 (5.0)	1 (2.6)	0.51
Percutaneous coronary intervention or bypass surgery	4 (10)	4 (10)	1.00
Dialysis vintage, months, median (IQR)	24 (15–42)	14 (9–28)	0.028
Cause of end-stage renal disease			0.052
Diabetes	16 (40)	9 (23)	
Hypertension	3 (7.5)	3 (7.7)	
Glomerulonephritis	12 (30)	8 (20)	
Polycystic kidney disease	2 (5.0)	9 (23)	
Interstitial nephritis	2 (5.0)	3 (7.7)	
Congenital anomalies	1 (2.5)	3 (7.7)	
Other/unknown	4 (10)	4 (10)	
Cardiovascular medications, *n* (%)			
Beta-blockers	19 (48)	21 (54)	0.37
ACE inhibitors	10 (25)	10 (26)	0.58
ARB	14 (35)	11 (28)	0.34
CCB	17 (43)	24 (62)	0.071
Diuretic	15 (38)	11 (28)	0.26
Statin	29 (73)	17 (44)	0.008
Fibrate	0 (0)	1 (2.6)	0.49
Ezetimibe	3 (7.5)	2 (5.1)	0.51
Aspirin	17 (43)	11 (28)	0.14
Blood pressure, mean (s.d.)			
Systolic blood pressure, mmHg	130 (29)	130 (18)	0.33
Diastolic blood pressure, mmHg	77 (13)	81 (12)	0.12
Heart rate, bpm, mean (s.d.)	72 (13)	75 (13)	0.56
Baseline serum measurements, median (IQR)			
Creatinine, μmol/L	715 (559–840)	787 (568–925)	0.38
N-Terminal brain natriuretic peptide, ng/mL	1487 (741–2535)	889 (554–1368)	0.28
Hemoglobin, g/L	114 (105–129)	119 (109–127)	0.54
C-reactive protein, ng/mL	2.9 (1.9–7.6)	1.5 (1.0–4.2)	0.019
Growth differentiation factor-15, pg/mL	5440 (4307–6452)	4744 (3639–5784)	0.13
PTH, pmol/mL	32 (18–67)	36 (19–79)	0.79
Cardiovascular magnetic resonance parameters, mean (s.d.)			
LVEDVi (mL/m^2^)	84 (22)	94 (24)	0.038
LVESVi (mL/m^2^)	34 (13)	40 (15)	0.043
LVEF (%)	59.8 (6.6)	57.6 (6.4)	0.13
LVMi (g/m^2^)	65.1 (20)	66.7 (20)	0.78
Cardiac index (L/min/m^2^)	3.49 (0.9)	3.96 (0.9)	0.024
GLS (%)	−16.6 (3.2)	−15.9 (3.0)	0.44
GCS (%)	−19.7 (3.6)	−18.1 (3.4)	0.057
GRS (%)	46.1 (13)	40.8 (11)	0.082

Abbreviations: *ACE* angiotensin converting enzyme; *ARB* angiotensin receptor blocker; *BMI* body mass index; *CCB* calcium channel blocker; *GLS* global longitudinal strain; *GCS* global circumferential strain; *GRS* global radial strain; *IQR* interquartile range; *LVEF* left ventricular ejection fraction; *LVESVi* left ventricular end-systolic volume index; *LVEDVi* left ventricular end-diastolic volume index; *s.d* standard deviation; *LVMi* left ventricular mass index; *PTH* parathyroid hormone

Compared to dialysis patients, KT patients were significantly younger (47 versus 56 years), with similar sex distribution and body mass index. There were no significant differences in cardiovascular risk factors, prior cardiovascular events, distribution of etiology for ESRD, or cardiovascular medications (with the exception of less statin use in KT group). At baseline, LVEDVi, LVESVi, and cardiac index were significantly higher in KT patients compared to dialysis patients, while no difference in LV strain parameters, LVEF, or LVMi was observed (Table 1).

Over the 12-month period, two myocardial infarctions and one cerebrovascular accident were observed in the dialysis group, while no cardiac event was observed in the KT group.

Table 2 shows the measured CMR parameters at baseline and at 12 months for dialysis and KT patients. When compared to baseline, mean LVEF for KT patients significantly improved at 12-month follow-up (57.6% ± 6.4% versus 60.7% ± 6.8%, *p* = 0.001), while no significant change was observed in dialysis patients (59.8% ± 6.6% versus 60.7% ± 5.6%, *p* = 0.40). Despite significant LVEF improvement compared to baseline for KT patients, comparison of change (from baseline to 12 months) between KT and dialysis patients did not reach statistical significance (mean difference − 2.5, 95% confidence interval [CI] -5.2-0.2, *p* = 0.070). The cardiac index at 12 months for dialysis and KT group were 3.6 ± 1.1 and 3.7 ± 0.8 L/min/m^2^, respectively, with no significant difference in the change from baseline between the two groups (mean difference 0.3, 95% CI -0.07-0.7, *p* = 0.10).

**Table 2:** Cardiovascular magnetic resonance parameters at baseline and 12 months for dialysis and kidney transplant patients

Cardiovascular magnetic resonance parameters, mean (s.d.)	Dialysis patients (*n* = 40)		Kidney transplant patients (*n* = 39)	
	Baseline	12 months	Baseline	12 months
LVEDVi (mL/m^2^)	84 (22)	84 (25)	94 (24)	82 (16)
LVESVi (mL/m^2^)	34 (13)	33 (13)	40 (15)	33 (10)
LVEF (%)	59.8 (6.6)	60.7 (5.6)	57.6 (6.4)	60.7 (6.8)
LVMi (g/m^2^)	65.1 (20)	63.9 (21)	66.7 (20)	61.2 (13.2)
GLS (%)	−16.6 (3.2)	−16.0 (3.2)	−15.9 (3.0)	−14.9 (3.0)
GCS (%)	−19.7 (3.6)	−19.7 (3.4)	−18.1 (3.4)	−19.4 (2.6)
GRS (%)	46.1 (13)	46.1 (12.5)	40.8 (11)	46.0 (9.5)

Abbreviations: *GLS* global longitudinal strain; *GCS* global circumferential strain; *GRS* global radial strain; *LVEF* left ventricular ejection fraction; *LVESVi* left ventricular end-systolic volume index; *LVEDVi* left ventricular end-diastolic volume index; *s.d* standard deviation; *LVMi* left ventricular mass index

**Fig. 1:**
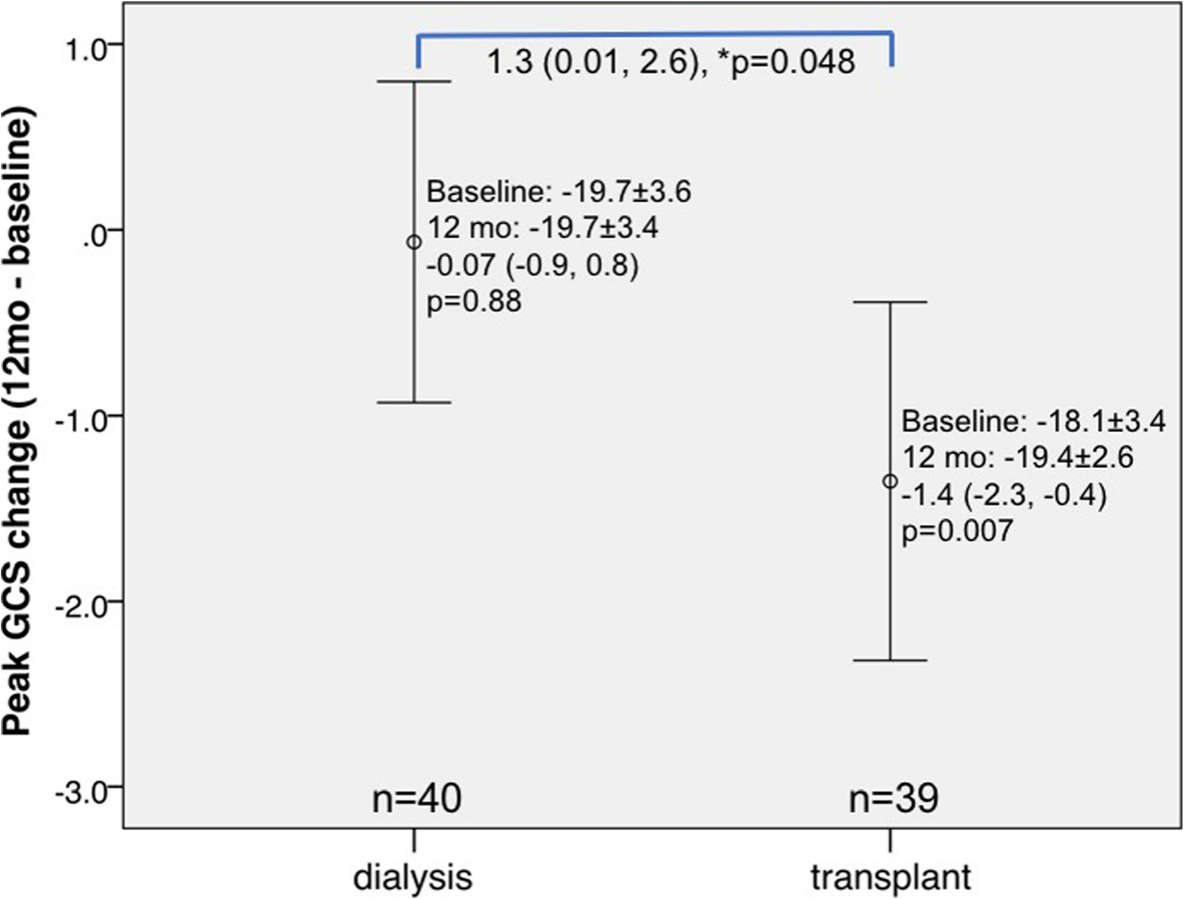
Changes in left ventricular strain parameter global circumferential strain assessed by cardiovascular magnetic resonance imaging at baseline and at 12-month in dialysis and transplant patients. *p denotes comparison of change (from baseline to 12 months) between kidney transplant (KT) and dialysis patients. Vertical bars denote 95% confidence intervals

**Fig. 2:**
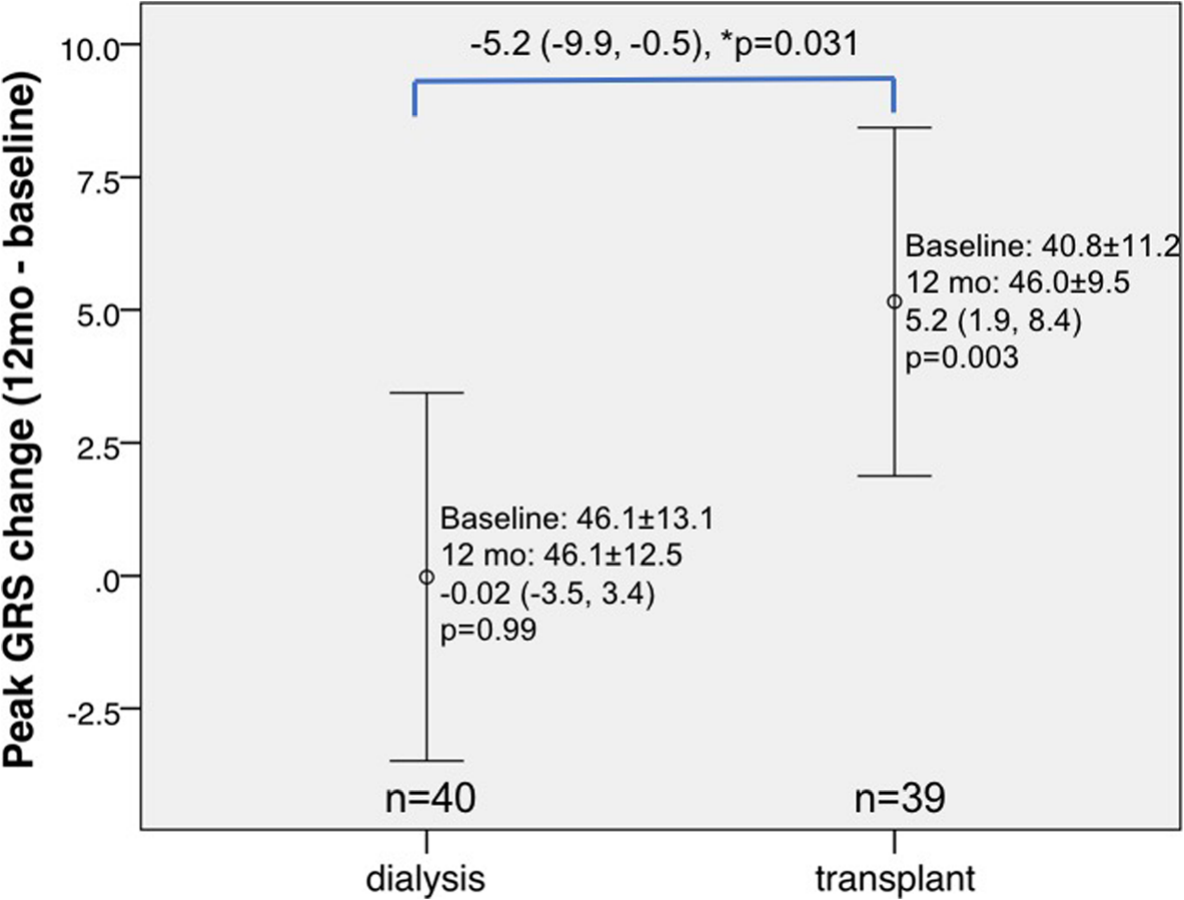
Changes in left ventricular strain parameter global radial strain assessed by cardiovascular magnetic resonance imaging at baseline and at 12-month in dialysis and transplant patients. *p denotes comparison of change (from baseline to 12 months) between KT and dialysis patients. Vertical bars denote 95% confidence intervals

**Fig. 3:**
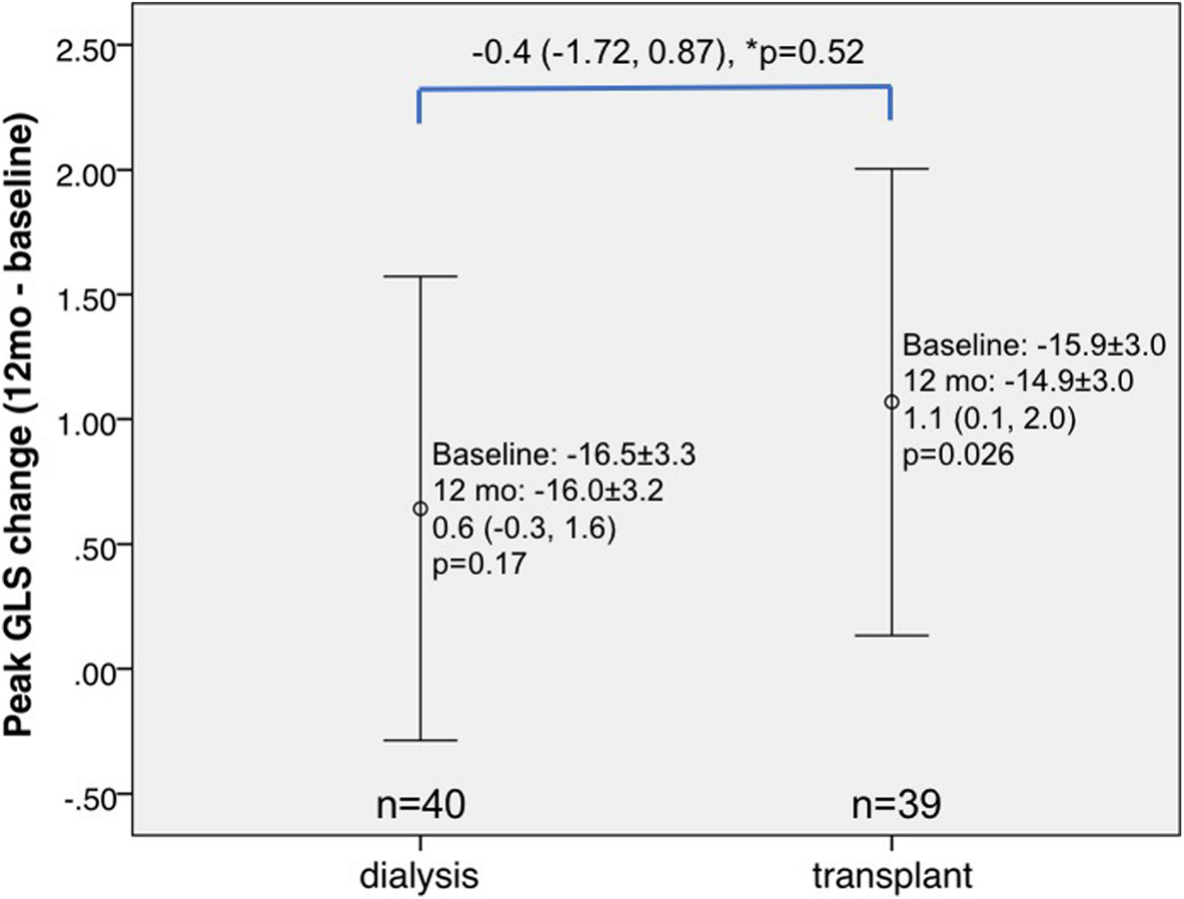
Changes in LV strain parameter global longitudinal strain assessed by CMR at baseline and at 12-month in dialysis and KT patients. *p denotes comparison of change (from baseline to 12 months) between KT and dialysis patients. Vertical bars denote 95% confidence intervals

Compared to baseline, KT group patients had significantly improved LV peak systolic strain parameters GCS (*p* = 0.007; Fig. 1) and GRS (*p* = 0.003; Fig. 2) at 12 months, while GLS was significantly worse (*p* = 0.026; Fig. 3). No significant improvement in LV strain parameters was observed for dialysis group patients. When comparing the 12-month changes between KT and dialysis patients, improvements in GCS (1.3, 95% CI -0.02-2.6, *p* = 0.048; Fig. 1) and GRS (− 5.2, 95% CI -0.5-9.9, *p* = 0.031; Fig. 2) were significant, while the decline in GLS was not (− 0.4, 95% CI -1.7-0.9, *p* = 0.52; Fig. 3). The intra-class correlation coefficients for intra-observer reproducibility were 0.91 (95% CI 0.77-0.96, *p*<0.001), 0.90 (95% CI 0.77-0.96, *p*<0.001), and 0.86 (95% CI 0.68-0.94, *p*<0.001), for GLS, GRS, and GCS, respectively.

Correlations between temporal changes in cardiac parameters and blood pressure are summarized in Table 3. For the entire cohort, there were significant correlations between change in LVEF and all three LV strain parameters from baseline to 12 months. We observed significant correlations between improvements in GCS and GRS with reductions in LVEDVi, and LVESVi, but not for GLS. At baseline, GLS was correlated with LVMi (Additional file 1). There was a significant weak positive correlation between changes in LVMi and GLS. These findings were similar for KT patients. We found no significant correlation between changes in LV strain parameters from baseline to 12 months and dialysis vintage (all *p* > 0.4, data not shown).

**Table 3:** Relationship between changes (from baseline to 12 months) in left ventricular peak systolic strain, ejection fraction, mass, volume, and blood pressure for the entire cohort

	LVEDVi	LVESVi	LVMi	LVEF	sBP	dBP
GLS	−0.001 (*p* = 0.99)	0.17 (*p* = 0.15)	0.26 (*p* = 0.020)	−0.34 (*p* = 0.002)	0.06 (*p* = 0.60)	0.09 (*p* = 0.43)
GCS	0.41 (*p* < 0.001)	0.52 (*p* < 0.001)	0.18 (*p* = 0.11)	−0.42 (*p* < 0.001)	0.19 (*p* = 0.11)	0.16 (*p* = 0.17)
GRS	−0.33 (*p* = 0.003)	−0.56 (*p* < 0.001)	− 0.12 (*p* = 0.30)	0.64 (*p* < 0.001)	− 0.07 (*p* = 0.57)	0.01 (*p* = 0.93)

Abbreviations: *dBP* diastolic blood pressure; *GLS* global longitudinal strain; *GCS*, global circumferential strain; *GRS* global radial strain; *LVEF* left ventricular ejection fraction; *LVESVi* left ventricular end-systolic volume index; *LVEDVi* left ventricular end-diastolic volume index; *LVMi* left ventricular mass index; *sBP* systolic blood pressure

At 12 months, the mean blood pressure for dialysis and KT patients were 135 ± 29/77 ± 13 and 126 ± 17/79 ± 11 mmHg, respectively. No correlation was observed between change in LV strain parameters and change in blood pressure (Table 3). The number of antihypertensive medications used was significantly less in the KT group at 12 months compared to baseline (2.4 ± 1.7 versus 1.5 ± 1.0, *p* = 0.001), while no difference was found for dialysis patients (2.1 ± 1.6 versus 2.1 ± 1.6, *p* = 0.54).

At 12 months, the median creatinine was 716 (IQR 580–894) and 108 (IQR 94–128) for the dialysis and KT groups, respectively. Following KT, there was no significant correlation between eGFR or creatinine with changes in LVEF, LVEDVi, LVESVi, LVMi, or systolic strain parameters (all *p* > 0.1) at 12 months.

We evaluated the association between biomarkers and LV strain at baseline in the overall cohort. At baseline, NT-BNP concentration was significantly correlated with LV strain parameters GLS (Spearman’s correlation coefficient 0.27, *p* = 0.019), GCS (Spearman’s correlation coefficient 0.38, *p* = 0.001), and GRS (Spearman’s correlation coefficient − 0.32, *p* = 0.005), suggesting that higher NT-BNP was associated with worse LV subclinical myocardial function. GDF-15 concentration was significantly correlated with LV strain parameter GLS (Spearman’s correlation coefficient 0.33, *p* = 0.003), but not GCS (Spearman’s correlation coefficient 0.088, *p* = 0.45) or GRS (Spearman’s correlation coefficient − 0.068, *p* = 0.56). CRP concentration was not correlated with LV strain parameters (all *p* > 0.1).

## Discussion

We conducted a prospective multi-centered cohort study in maintenance dialysis patients who were KT candidates to evaluate LV function changes after KT using FT-CMR strain imaging. At 12-month post-KT, we observed significant improvements in key parameters of LV strain (GCS and GRS), with a concurrent improvement in LVEF. This was in contrast to the lack of change observed in these parameters for patients who remained on dialysis. To our knowledge, this is the first study to evaluate myocardial strain by CMR in ESRD patients before and after KT. These findings support the notion that KT has favorable effects on LV function, and highlight the utility of CMR strain to detect subclinical improvements in myocardial function following KT.

In this study, both dialysis and KT patients had generally preserved LVEF at baseline. This is not surprising given the fact that LVEF represents late LV dysfunction [8, 23], and is consistent with previous studies demonstrating preserved LVEF using echocardiography in a large proportion of ESRD patients [5–7]. Although LVEF significantly improved from baseline to 12 months in the KT group, the change only trended towards significance when compared to dialysis patients. This may be attributed to the fact that most patients had preserved LVEF before KT, and possibly a selection bias since patients considered to be KT-eligible represent the healthiest subset of the dialysis population. These findings are consistent with studies by Wali et al. and Casas-Aparicio et al. demonstrating improved LVEF following KT [24, 25].

Although echocardiography is more accessible for strain imaging, its accuracy is limited by the adequacy of acoustic windows, image quality, and operator-dependent variability. Furthermore, dialysis-associated fluctuations in intravascular volume and intracardiac volume likely further compromise the reliability and accuracy of ventricular function indices by echocardiography [26]. As such, CMR is the gold standard to provide accurate and reproducible measurements of volume and mass due to lack of geometric assumptions, and less load-dependence. CMR with myocardial tagging is currently the reference standard technique for myocardial deformation. Strain can be measured by harmonic phase analysis (HARP) and spatial modulation of magnetisation (SPAMM) [27, 28], allowing detection of LV dysfunction even in asymptomatic subjects without cardiovascular disease [29]. The novel FT-CMR technique for measuring strain using bSSFP sequence, unlike myocardial tagging, requires no additional sequences as the cine images required are part of the routine LV study protocol, allowing for rapid acquisition and post-processing. Moreover, FT-CMR has been validated against myocardial tagging using HARP and SPAMM for systolic and diastolic strain [30, 31]. In addition, it is important to highlight that the advantage of strain over LVEF is that it is less sensitive to load changes. Accordingly, in this study, we measured LV peak systolic strain GLS, GCS, and GRS by FT-CMR to assess the impact of KT on systolic function.

While we demonstrated a significant improvement in GCS and GRS after KT when compared to dialysis patients, no improvement in GLS was observed. These changes in GRS and GCS were correlated with changes in LVEF. Of the strain parameters measured by speckle tracking echocardiography, consensus recommendation favoured use of GLS for early detection of subclinical LV dysfunction [32]. This is because GLS has been reported to precede clinical evidence of overt systolic dysfunction in a variety of cardiomyopathies [33, 34]. Similarly, in the ESRD population, a recent study by Hensen et al. demonstrated a high prevalence of impaired GLS (measured by echocardiography) in pre-dialysis and dialysis patients with preserved LVEF, and that impaired GLS was an independent risk factor for HF and mortality. Our strain results are in contrast with Hewing et al., which showed improvement in GLS post-KT as assessed by echocardiography in a population of patients with preserved LVEF at baseline [35]. The precise reason for the discrepant findings is unclear, and may be partly attributed to different imaging modality used. Moreover, the reason for improvement in GCS and GRS but not GLS is elusive. It is plausible that the improvement in LVEF may be due to improvement in GCS and GRS compensating for abnormal GLS. Prior studies examining LV strain parameters demonstrated that GLS deteriorates in early stages of myocardial pathologic conditions, before reduction in LVEF, while GCS remain preserved or increased to compensate for GLS function [36–38]. There are no prior studies specifically investigating the temporal sequence of deterioration or improvement of cardiac strain following a cardiovascular intervention. In patients who received cardiac resynchronization therapy and aortic valve replacement, some studies demonstrated that improvement in GCS rather than GLS is crucial for favorable remodeling, while others demonstrated improvement in both GCS and GLS [38–43]. Hence, it is also plausible that GCS and GRS are more sensitive to the effects of treatment (KT) and evolve before GLS. We also note that GCS is the most reproducible strain parameter by FT-CMR [21]. To the best of our knowledge, no prior studies investigated the temporal sequence of deterioration or improvement of cardiac strain in this setting. As highlighted above, studies have implicated abnormal GLS as a predictor of worse prognosis in CKD and dialysis patients [6, 14, 44], which may be secondary to myocyte hypertrophy and microvascular ischemia due to myocardial fibrosis [45]. Hence, the long-term implications for lack of improvement in GLS following KT are unclear and need to be addressed in future studies with longer follow-up CMR.

We examined the relationships between LV strain parameters and LV volumes and blood pressure to determine whether a relationship exists between structural and functional changes. We found improvements in GCS and GRS, despite a concurrent reduction in LV volumes LVEDVi (surrogate of preload) and LVESVi. The correlation between improvement in LV strain with decreased LV volume suggest that improved LV systolic function is likely attributed to KT rather than loading changes. Similarly, evaluation of blood pressure changes is required for interpretation of LV function change. Afterload is the tension or stress generated in the LV wall during myocardial contraction to eject blood. An assumption can be made such that afterload is proportional to the aortic pressure that the LV must overcome to eject blood, with systolic blood pressure being a surrogate of afterload. In this study, we did not observe a significant change in blood pressure following KT compared to baseline, and there was no correlation with LV strain. Interestingly, KT patients required significantly fewer antihypertensive medications at 12 months, while number of medications remained similar in dialysis patients. Taken together, our findings show that reduction in LVEDVi and LVESVi at 12 months occurred without blood pressure change, suggesting that strain improvements were not simply a result of changes in load.

Patients with advanced CKD frequently have increased LVM, which is further exacerbated by the receipt of dialysis [46, 47]. In trials of dialysis intensification, LVM served as a well-established surrogate endpoint for adverse cardiovascular events [48]. Although LVM regression has been previously evaluated [49], there is currently a knowledge gap as to whether LV functional change is associated with structural change. Evaluating change in LV function using strain parameter post-transplant is imperative in light of recent studies supporting the role of myocardial strain as an independent predictor of CKD mortality [14, 15]. Taken together, our study is one of the first to address this key question, with findings suggestive of systolic function improvement 12 months after KT.

We found no correlation between changes in LVEF or LV strain parameters with changes in renal function as reflected by eGFR and creatinine at 12 months post-transplant. These data do not provide insight into whether mitigation of uremia is a potential mediator for LV functional changes observed following transplant. Furthermore, although creatinine is a measure of renal function, creatinine alone likely does not adequately reflect all the beneficial cardio-renal effects. The reduction in mortality is likely related to myriad of metabolic improvements that result in favourable effects on cardiac function, including improvement in anemia, calcium-phosphate profile, reduction of parathyroid hormone, and neurohormones [50].

Our study has a number of strengths. There are limited data on CMR-derived strain in CKD and KT patients. To the best of our knowledge, the present study is the first to examine whether systolic strain by FT-CMR is a useful tool for identifying improvement in LV systolic function following KT. Advantages of CMR include greater reproducibility compared to echocardiogram, particularly pertaining to LVM and LV volume whereby patients on dialysis (i.e. control group in our study) experience greater fluctuations in volume. CMR was performed using different vendors at 3 centres, enhancing the generalizability of our results. CMR analyses were completed in a blinded fashion and serial CMRs were analyzed in random order. Our study reported temporal changes in systolic strain at 12 months after KT, providing one of the longest longitudinal follow-up in the literature. In addition, our results support the notion that KT improves LV contractility over time. Although the precise pathophysiological reasons for improved cardiovascular outcomes following KT compared to dialysis remain to be clarified, it is plausible that survival benefit is at least in part due to amelioration of metabolic derangements and efficient clearance of numerous uremic toxins that may be cardiotoxic [51] and have negative inotropic effects [52–54].

This study has a number of evident limitations. Since this was not a randomized trial, the relationship between KT and various strain parameters cannot be viewed as causal, thus causality cannot be established from our results. However, randomized trials of KT are logistically very challenging to conduct. Secondly, we could not evaluate changes in strain long term beyond our study period (> 12 months) and longer follow-up is needed to assess whether strain improvements are sustained following KT. Our study may lack power in identifying important inter-group differences due to the relatively small number of patients. We were not able to provide a precise reason for deterioration of strain parameter GLS which was discordant with the improvements in GCS and GRS. Our study was not designed or powered sufficiently to address the prognostic role of GLS versus GCS or GRS in KT patients. Future studies are required to address the temporal nature of improvement of the 3 strain parameters in KT patients. While immunosuppressive agents used in KT patients (steroids and calcineurin inhibitors) may exacerbate cardiovascular disease for a variety of reasons, our study was not designed or adequately powered to definitively determine the effect of immunosuppressive regimens on myocardial function, which would be better addressed in a separate randomized study. Our study sample size may have been underpowered to detect associations between CMR parameters and blood pressure. We did not measure myocardial strain by echocardiogram and could not determine the correlations between strain measurements measured by different imaging modalities. Given our small sample size, together with limited follow-up of 1-year timeframe and low number of cardiovascular events observed, our study is ill-equipped to determine the incremental prognostic value of strain, although our measured CMR parameters are known to be important surrogates for clinical events in diverse cardiovascular conditions. Finally, other CMR parameters, such as diastolic function, are beyond the scope of this study.

## Conclusion

In this prospective longitudinal study comparing KT patients with those who remained on dialysis, we observed a significant improvement in LV systolic strain GCS and GRS at 12 months, but not GLS, with corresponding improvement in LVEF and LV volumes. Our results support the notion that KT likely has favourable effects on LV structure and function. Additional studies are required to confirm these findings in a larger cohort of KT patients with longer follow up.

## Additional file

Additional file 1: **Figure S1.** Scatter plot of the correlation between baseline global longitudinal strain and left ventricular mass indexed to body surface area. (DOCX 4798 kb)

## Abbreviations

BMI: Body mass index
bSSFP: Balanced steady state free precession
CI: Confidence interval
CKD: Chronic kidney disease
CMR: Cardiovascular magnetic resonance
CRP: C-reactive protein
EDV: End diastolic volume
EDVi: End diastolic volume index
eGFR: Estimated glomerular filtration rate
ESRD: End-stage renal disease
ESV: End systolic volume
ESVi: End systolic volume index
FT: Feature tracking
GCS: Global circumferential strain
GLS: Global longitudinal strain
GRS: Global radial strain
HARP: Harmonic phase analysis
HF: Heart failure
IQR: Interquartile range
KT: Kidney transplant
LV: Left ventricle/left ventricular
LVEDV: Left ventricular end-diastolic volume
LVEF: Left ventricular ejection fraction
LVESV: Left ventricular end-systolic volume
LVH: Left ventricular hypertrophy
LVM: Left ventricular mass
LVMi: Left ventricular mass index
NT-BNP: N-terminal brain natriuretic peptide
SPAMM: Spatial modulation of magnetization
