# Supplementary material for: Cardiovascular magnetic resonance left ventricular strain in end-stage renal disease patients after kidney transplantation
Source: J Cardiovasc Magn Reson. 2018 Dec 17;20:83. doi: 10.1186/s12968-018-0504-5 (PMC6296102; doi:10.1186/s12968-018-0504-5)
Supplement: Supplementary file 1 — Figure S1. Scatter plot of the correlation between baseline global longitudinal strain and left ventricular mass indexed to body surface area. (DOCX 4798 kb) [file 12968_2018_504_MOESM1_ESM.docx]

Supplementary File 1


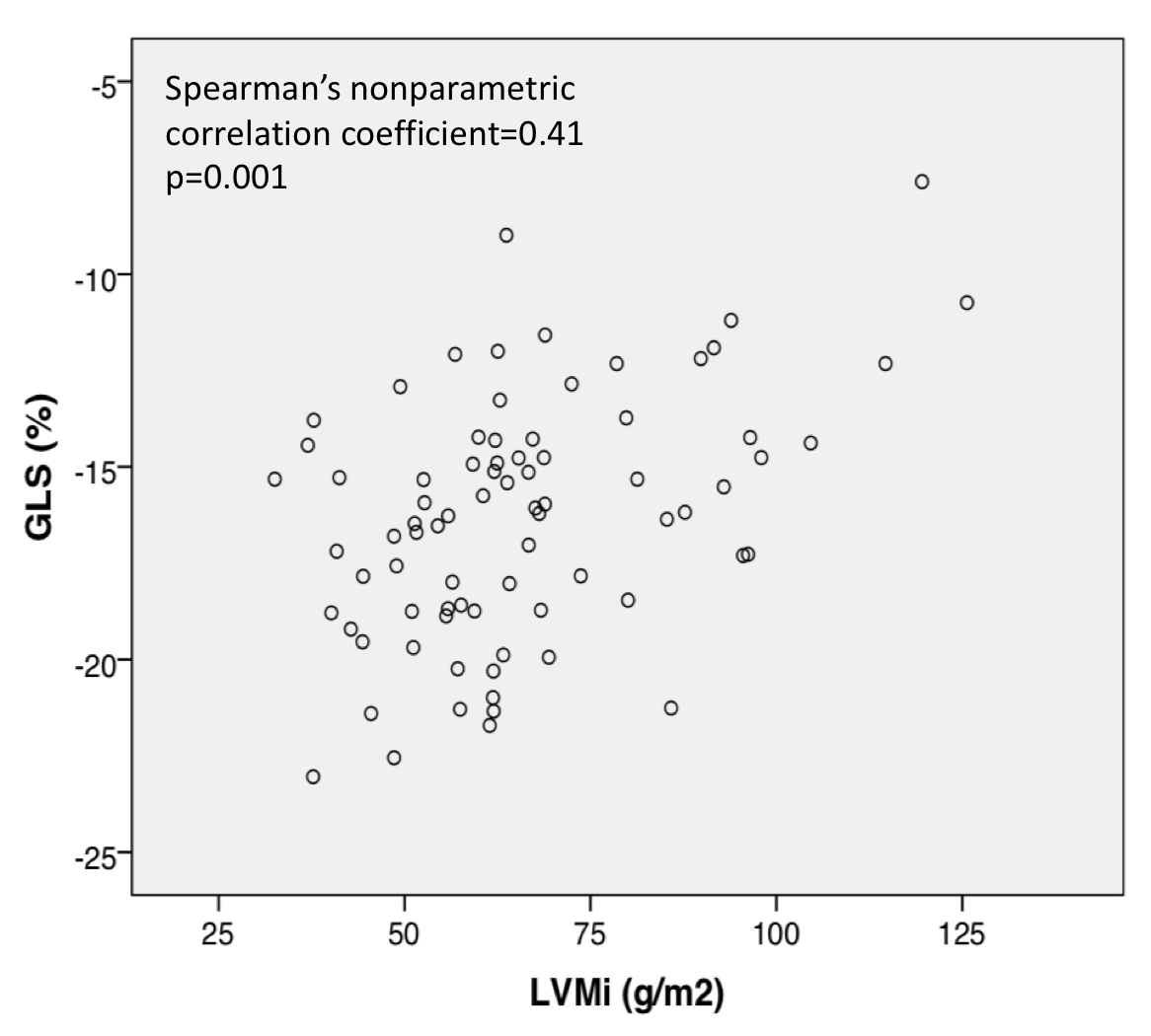


**Supplementary Figure** Scatter plot of the correlation between baseline global longitudinal strain and left ventricular mass indexed to body surface area.
